# Ticagrelor combined with aspirin may improve patency of vein graft one year after off-pump coronary artery bypass grafting: a single-center, randomized double-blinded clinical controlled trial

**DOI:** 10.3389/fcvm.2025.1461370

**Published:** 2025-05-09

**Authors:** Wenjun Wang, Qingzhuo Chi, Feng Gao, Xuezhi He, Yang Gao, Lei Shi, Wei Liu, Weiwang Fan, Lin Zhang, Chang Xu, Xijing Zhuang

**Affiliations:** ^1^ Department of Cardiovascular Surgery, Dalian Municipal Central Hospital, Dalian, China; ^2^ School of Energy and Power, Dalian University of Technology, Dalian, China

**Keywords:** coronary artery bypass surgery, off-pump, saphenous vein graft, ticagrelor, clopidogrel

## Abstract

**Background:**

Dual anti-platelet therapy (DAPT) after coronary artery bypass graft surgery (CABG) has drawn a lot of controversy. This study aimed to explore the effects of ticagrelor combined with aspirin (compared with aspirin combined with clopidogrel) on the patency of saphenous vein graft (SVG) after off-pump CABG.

**Methods:**

This was a prospective, randomized, double-blinded clinical controlled trial. Participants were first given aspirin (100 mg/d) within 12 h after off-pump CABG, followed by P2Y12 receptor antagonist (Orally, 75 mg/time of clopidogrel, once daily, for Group C and 90 mg/time of ticagrelor, twice daily, for Group T) within 24 h after off-pump CABG for one year. Computed tomography angiography (CTA) was conducted for all patients. The incidence of major adverse cardiac events(MACE), death, stroke, hemorrhage, left ventricular diameter (LVD), and left ventricular ejection fraction (LVEF) of the participants was assessed one year after off-pump CABG based on a 12-month follow-up.

**Results:**

A total of 73 participants completed the follow-up, and 219 bypass grafts, including 146 SVGs, were conducted. Notably, 11 bypass grafts (SVGs) were exposed to occlusion (9 in Group C and 2 in Group T). The overall occlusion rates of bypass grafts and SVGs of Groups C and T were significantly different (9/114 vs. 2/105, *P* = 0.043, 9/76 vs. 2/70, *P* = 0.04). Moreover, multivariate binary Logistic regression demonstrated that ticagrelor + aspirin anti-platelet therapy could reduce the stenosis risk of bypass grafts (OR = 0.195, 95% CI = 0.039-0.978, *P* = 0.047).

**Conclusions:**

Compared with clopidogrel, ticagrelor may reduce the occlusion rate of vein grafts after CABG.

**Clinical Trial Registration:**

[https://www.chictr.org/], identifier [ChiCTR1900022390].

## Background

Coronary artery bypass graft surgery (CABG) is widely used for coronary heart disease treatment (1). Several patients have undergone off-pump CABG due to the development of clinical technology (2). Bypass vessel transplantation mainly involves the combination of the left internal mammary artery (LIMA) and the great saphenous vein (3).

**Figure 1:**
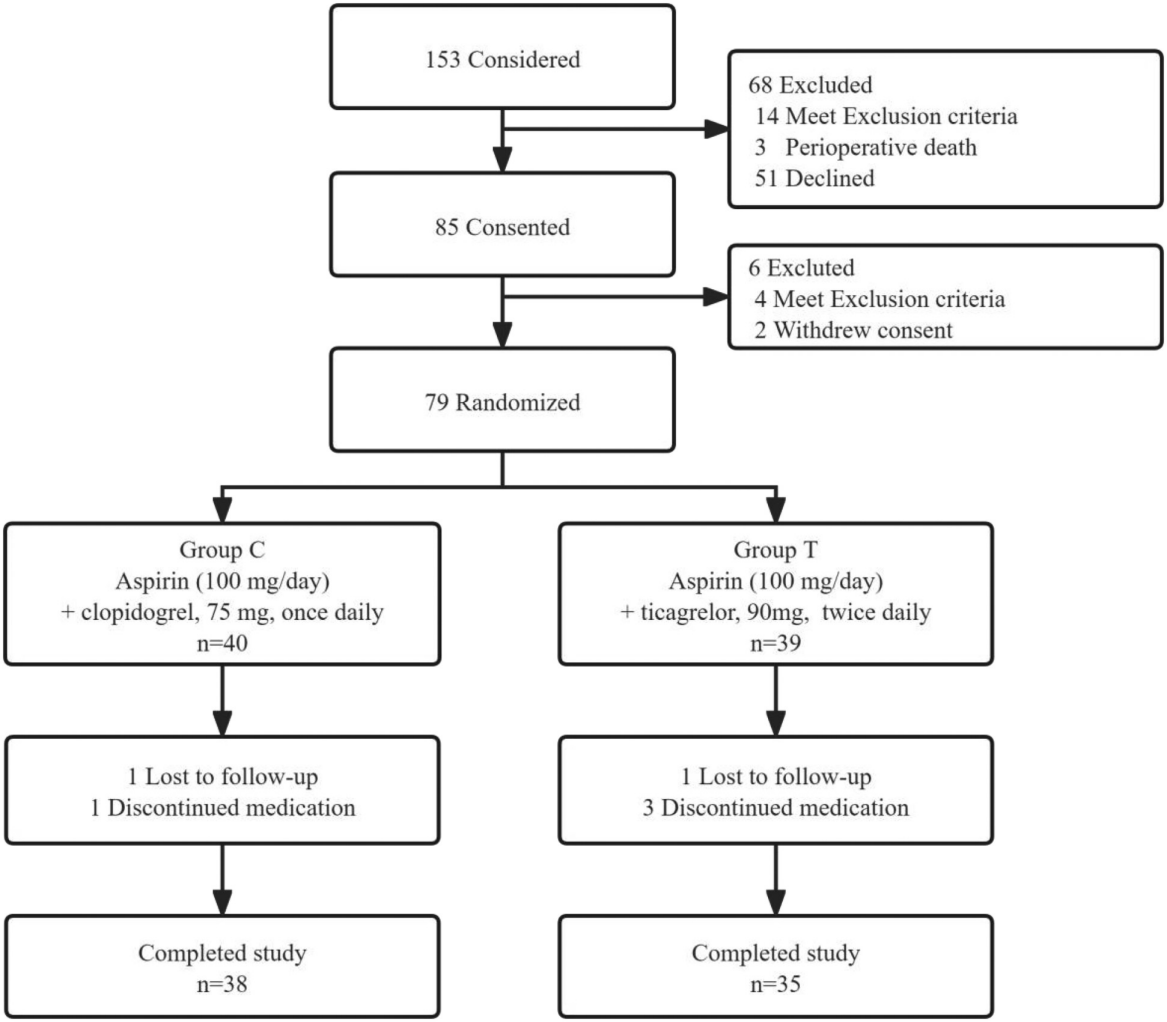
Flow chart of the study.

However, some patients may experience ischemic attack after CABG treatment, mainly due to SVG stenosis/occlusion. About 26.6%, 25.0%, and 1.5% of target vessels in patients undergoing PCI are bypass grafts after CABG, great SVG, and arterial grafts, respectively (4). Therefore, SVG patency should be improved to enhance cardiovascular surgery.

Studies have shown that the activated platelet system is associated with occlusion of bypass grafts. Notably, studies have shown that aspirin, a traditional anti-platelet drug, can improve the patency of bypass grafts after CABG surgery (5). Moreover, studies have shown that P2Y12 receptor antagonist combined with aspirin has synergistic anti-platelet effects. As a result, the dual anti-platelet therapy (DAPT) of aspirin combined with P2Y12 receptor antagonist has been widely applied in secondary prevention after CABG surgery (6). Clopidogrel, a classic P2Y12 receptor antagonist, has been used for many years clinically. However, ticagrelor, as an alternative P2Y12 receptor antagonist, has led to some controversies regarding the development of DAPT strategies.

Ticagrelor has a faster onset and better efficacy than Clopidogrel. Besides, several PLATO trials have shown that Ticagrelor has better efficacy than Clopidogrel (7, 8). Saw et al. found that DAPT involving ticagrelor can better maintain the patency of SVG than aspirin alone (9). Subsequent DACAB trials also showed similar findings through multi-center clinical trials (10). Nevertheless, the Popular-CABG trials found that the DAPT involving ticagrelor cannot maintain better patency of SVG compared with aspirin (11).

The American Heart Association suggested that the DAPT containing ticagrelor combined with aspirin may be superior to clopidogrel combined with aspirin (Class IIa, Evidence Level B) for secondary prevention after CABG surgery (7). Although the European guidelines do not recommend using ticagrelor for DAPT in stable CAD patients after CABG, they suggest performing DAPT under safe conditions (12).

This randomized controlled trial aimed to verify whether ticagrelor combined with aspirin can improve the patency of saphenous vein graft (SVG) after off-pump CABG (compared with aspirin combined with clopidogrel).

## Methods

### Study design and ethics approval

This was a single-center, prospective, randomized, double-blinded clinical controlled trial, following the principles of the Helsinki Declaration and all applicable laws and regulations (13).

Only patients who received simple CABG were included in this study. The patients were randomly divided into two groups: the aspirin + ticagrelor group and the aspirin + clopidogrel group. The patients provided written informed consent before participation. The study was approved by the Ethics Committee of Dalian Central Hospital (Ethics approval number: YN-2019-018-01) and registered at the Clinical Trial Center in China (Registration no. ChiCTR1900022390. Date of first registration: 09/04/2019). The study follows the CONSORT guidelines for reporting randomised clinical trials.

### Clinical setting and participants

Only patients aged 18–80 years who underwent selective simple off-pump CABG at our center with intraoperative bypass grafts of LIMA-left anterior descending coronary artery (LAD), SVG-left circumflex artery (LCX), and SVG-right coronary artery (RCA) were enrolled in this study. Exclusion criteria included: (1) patients who did not receive CABG; (2) patients who underwent other cardiac surgeries at the same period; (3) patients who received emergency CABG; (4) patients with contraindications to clopidogrel or ticagrelor; (5) patients with sinus bradycardia; (6) patients simultaneously receiving liver enzyme potent inducer or inhibitor treatment; (7) patients who required kidney replacement therapy.

All patients underwent transthoracic echocardiography upon admission for evaluation of the structure and function of the left ventricle. The patients received aspirin until the day of CABG surgery. Notably, all patients had signed informed consent before CABG surgery. All patients survived after the CABG surgery, and no contraindications to aspirin/ticagrelor/clopidogrel were observed. Patients were randomized using the random-number table system of the Clinical Trial Center in China. The patients were randomly assigned to the experimental group (Group T) and control group (Group C). The age, gender, height, weight, BMI, coronary heart disease status at admission, previous PCI history, surgical history, hypertension history, diabetes history, smoking history, drinking history, left ventricular diameter and left ventricular ejection fraction (LVEF) were also recorded.

The patients received aspirin (100 mg/day) within 12 h after CABG surgery, followed by P2Y12 receptor antagonist (ticagrelor/clopidogrel, orally) within 24 h after surgery, for 1 year. Patients in the experimental group received ticagrelor, 90 mg, twice daily, while those in Group C received clopidogrel, 75 mg, once daily.

Other heart medications were determined by the therapists. Patients were assigned unique random codes via the Resman system program from the Chinese Clinical Trial Registry Center. Randomization was performed using a computer-generated random number method, and the allocation results were sealed in opaque envelopes by the research coordinator for secure preservation. The research drugs were distributed by cardiologists based on random coding. The study coordinators, patients, physicians, and computed tomography angiography (CTA) interpreters were blinded to drug distribution.

The patients were monitored daily during hospitalization. Moreover, the patients were followed up at 3, 6, and 12 months after discharge. Cardiac CTA and echocardiography examinations were performed one year after surgery.

### Variables and measurements

#### Patency indicators of bypass grafts

The 64-detector rows of coronary artery CTA were re-examined after one year of surgery to evaluate the patency of coronary artery bypass grafts. Technician conducting CTA examination was blinded to the anti-platelet drugs (only aware of the patient's CABG postoperative condition). The degree of coronary artery stenosis was defined based on the FitzGibbon grading, where A and B grades were defined as the patent bypass grafts (14).

#### Major adverse cardiac events (MACE)

MACE was defined as cardiac death, recurrent myocardial infarction, new-onset agina, and cardiac shock and were observed during the follow-up (15).

#### Hemorrhage incidence

The main hemorrhage incidents were defined as life-threatening hemorrhage incidents (fatal hemorrhage, intracranial hemorrhage, hemorrhage requiring surgical intervention, hemorrhage leading to severe hypotension, and hemorrhage requiring venous vasoactive drugs), fundus bleeding, hemoglobin reduction exceeding 5 g/d, or blood transfusion exceeding 2 U during the treatment (due to hemorrhage incidents). Minor bleeding was defined as a bleeding event that did not lead to the main hemorrhage incidents (16).

#### Left ventricular structure and function

The LVEF value and the left ventricular end-diastolic diameter were assessed via cardiac ultrasound at the admission and at the end of follow-up.

#### Other indicators

Other indicators included incidences of death and stroke during follow-up.

### Statistical analysis

Sample size estimation: PASS 15.0 was used for sample size estimation. Literature has reported that the patency rate of venous grafts in patients taking clopidogrel and aspirin was 91.6% three months post-CABG. However, approximately six months post-surgery, the patency rate of venous grafts decreased to around 87% (17, 18). Based on the literature we have reviewed and our clinical observations, we estimate that the patency rate of vein bypass grafts in Group C will be 80% one year post-CABG. Based on relevant studies in recent years, we have found that patients treated with ticagrelor and aspirin have a patency rate of over 90% for vein grafts three months to one year after CABG (11). Moreover, with the advancement of surgical techniques, the patency rate of vein grafts is further improving (19). Therefore, the vein graft patency of Group T after one year of surgery was estimated to be 95%. The sample size of 146 bypass grafts was required to detect an absolute difference of 5% with 80% statistical power and 0.05 bilateral a. Notably, 73 patients were needed for random assignment.

Statistical analysis: the general information and follow-up results of patients were imported into Office 365. SPSS 26.0 was used for all statistical analyses. Measurement data were expressed as mean ± standard deviation while counting data were expressed as frequency and percentage. The t-test and *χ*^2^ test were used for inter-group comparison of measurement data and inter-group comparison of counting data, respectively. The counting data with an expected frequency of less than 5 underwent Fisher's exact test. Univariate analysis was conducted to evaluate the impact of antiplatelet therapy strategies, gender, age, height, weight, body mass index (BMI), systolic and diastolic blood pressure, heart rate, respiratory rate, smoking history, hypertension history, diabetes history, percutaneous coronary intervention (PCI) history, surgical history, left ventricular end-diastolic diameter (LVD) at admission, and left ventricular ejection fraction (LVEF) at admission on venous graft patency. Subsequently, multivariate logistic regression analysis was performed to determine the independent effects of these factors on venous graft patency. All tests were conducted bilaterally, with *P* < 0.05 indicating a statistically significant difference.

## Results

### Participants, descriptive data, and outcome data

Patient recruitment started from April 2019 to April 2021. but was terminated prematurely due to coronavirus disease 2019 (COVID-19). A total of 153 patients underwent CABG alone in our center and received bypass grafts of LIMA-LAD, SVG-LCX, and SVG-RCA. Finally, 85 patients participated in this study, of which six were excluded after CABG due to contraindications or withdrawal of the consent form. The remaining 79 patients were randomly assigned to Groups C and T. Four patients discontinued medication within three months after surgery due to condition changes. Moreover, two patients were lost during follow-up, and 73 patients completed the follow-up (Figure 1). The general parameters collected from the patients included gender, age, height, weight, body mass index (BMI), systolic and diastolic blood pressure upon admission, resting heart rate, respiratory rate, coronary heart disease status at admission (including stable angina pectoris, unstable angina pectoris, or non-ST-segment elevation myocardial infarction), history of previous surgeries, prior percutaneous coronary intervention (PCI) history, presence of hypertension, preoperative left ventricular internal diameter, and preoperative left ventricular ejection fraction. Notably, the baseline characteristics were not significantly different between the two groups (Table 1).

**Table 1:** Comparison in general parameters between groups C and T.

Parameters	Group C (*n* = 38)	Group T (*n* = 35)	Statistical value	*P*
Male (cases)	26	28	1.269^a^	0.260
Female (cases)	12	7	–	–
Age (years)	65.13 ± 7.85	66.05 ± 7.57	0.512^b^	0.611
Height (cm)	169.89 ± 8.24	169.34 ± 7.51	0.298^b^	0.767
Weigh (kg)	71.87 ± 10.30	71.03 ± 9.03	0.369^b^	0.713
BMI	24.87 ± 2.87	24.77 ± 2.69	0.153^b^	0.879
Systolic Blood Pressure (mmHg)	140.00 ± 21.95	136.80 ± 21.86	0.624^b^	0.535
Diastolic Blood Pressure (mmHg)	80.53 ± 9.87	77.77 ± 11.40	0.902^b^	0.370
Respiratory Rate	18.74 ± 2.33	19.03 ± 2.42	0.524^b^	0.602
Resting Heart Rate	75.42 ± 15.57	77.77 ± 11.40	0.763^b^	0.448
Presentation	–	–	0.259^a^	0.875
Stable angina	12	13	–	–
Unstable angina	21	18	–	–
NSTEMI	5	4	–	–
Previous surgical history	15	13	0.042^a^	0.838
Previous PCI	5	3	^c^	0.712
Smoke(cases)	23	15	2.779^b^	0.131
Diabetes (cases)	14	18	1.574^b^	0.210
Hypertension (cases)	25	25	0.268^b^	0.604
Left ventricular internal diameter	51.53 ± 6.08	51.74 ± 7.57	0.135^b^	0.893
Left ventricular ejection fraction	54.55 ± 11.62	53.91 ± 13.91	0.213^b^	0.832

^a^ *χ*^2^ test.

^b^ Student's *t* test.

^c^ Fisher's exact test.

### Main results

A total of 219 bypass grafts were evaluated, of which 73 grafts were LIMA-LAD. Moreover, 146 SVGs were detected, of which 73 grafts were SVG-LCX, and 73 grafts were SVG-RCA. CTA examination revealed that 11 bypass grafts (SVGs) were exposed to occlusion. Nine bypass grafts in Group C, including six SVG-LCX and three SVG-RCA, were exposed to occlusion. Two bypass grafts in Group T (SVG-RCA) were exposed to occlusion. The total occlusion rate of bypass grafts and SVGs were significantly different between Groups C and T (9/114 vs. 2/105 *P* = 0.043, 9/76 vs. 2/70, respectively *P* = 0.04) (Table 2).

**Table 2:** Graft occlusion and stenosis rates according to randomised treatment.

Parameters	Group C	Group T	Statistical value	*P*
Total graft occlusion	9	2	4.111^a^	0.043^a^
SVG graft occlusion	9	2	4.223^a^	0.04^a^
SVG-LCX	6	2	^b^	0.264
SVG-RCA	3	0	^b^	0.241

^a^ *χ*^2^ test.

^b^ Fisher's exact test.

One patient in Group C developed a stroke during the follow-up. Moreover, 16 patients had hemorrhage incidents in Group T, of which two had major bleeding events. Both of the 2 patients with major bleeding events had gastrointestinal bleeding. The incidence of minor bleeding incidents was significantly higher in Group T than in Group C (4/38 vs. 10/35 *P* = 0.05). Seven patients developed MACE, of which one patient in Group C developed NSTEMI, and all other patients developed new-onset angina. Notably, MACE was not significantly different between the two groups (5/38 vs. 2/35 *P* = 0.432) (Table 3).

**Table 3:** Clinical events between groups C and T.

Parameters	Group C (*n* = 38)	Group T (*n* = 35)	Statistical value	*P*
Minor Bleeding	4	10	3.827^a^	0.05
Major Bleeding	0	2	^b^	0.226
MACE	5	2	^b^	0.432
Angina	4	2	^b^	0.676
NSTEMI	1	0	^b^	1.000
Stroke	1	0	^b^	1.000

^a^ *χ*^2^ test.

^b^ Fisher's exact test.

Nonetheless, LVD and LVEF of cardiac ultrasound were not significantly different between Groups C and T (Table 4).

**Table 4:** Left ventricular diameter and left ventricular ejection fraction 1 year after surgery.

Parameters	Group C (*n* = 38)	Group T (*n* = 35)	Statistical value	*P*
Left ventricular internal diameter	50.79 ± 4.45	50.40 ± 7.52	0.266	0.791
Left ventricular ejection fraction	57.05 ± 8.75	55.46 ± 10.50	0.707	0.482

The univariate predictive factors for occlusion of bypass grafts are shown in Table 5. Ticagrelor + aspirin DAPT use predicted a significantly lower graft occlusion(OR 0.035, 95% CI 0.002–0.570, *p* = 0.018). The predictive factors of vein graft patency were analyzed using multivariate binary Logistic regression via stepwise regression analysis. Finally, the regression model suggested that ticagrelor + aspirin DAPT could reduce the stenosis risk of bypass grafts (OR = 0.195, 95% CI = 0.039-0.978, *P* = 0.047).

**Table 5:** Univariate predictors of graft occlusion.

Parameters	OR	95%CI	*P*
Ticagrelor	0.195	0.039–0.978	0.047
Male	0.997	0.02–49.35	0.999
Age	0.972	0.842–1.122	0.695
BMI	10.312	0.074–1430.476	0.354
Height (cm)	1.74	0.39–7.76	0.468
Weigh (kg)	0.461	0.083–2.563	0.376
Systolic Blood Pressure (mmHg)	0.994	0.94–1.052	0.844
Diastolic Blood Pressure (mmHg)	0.865	0.721–1.039	0.122
Resting Heart Rate	1.018	0.946–1.094	0.637
Respiratory Rate	1.043	0.642–1.694	0.864
Left Ventricular Internal Diameter	1.274	0.99–1.639	0.06
Left Ventricular Ejection Fraction	1.096	0.971–1.237	0.136
Previous surgical history	1.191	0.155–9.128	0.867
Previous PCI	0	0	0.999
Smoke (cases)	0.417	0.109–1.59	0.2
Hypertension (cases)	0.741	0.078–7.048	0.794
Diabetes (cases)	1.78	0.184–17.219	0.618

## Discussion

SVG occlusion depends on the following three pathophysiological processes after CABG surgery: thrombosis and technical failure are the main mechanisms in the first week and month after CABG, followed by intimal hyperplasia from one month to one year, and atherosclerosis one year later.

Intimal hyperplasia of SVG is an adaptive mechanism for high arterial pressure (“arterialization”). The activation of the platelet system plays an essential role in the arterialization process (20). Activated platelets secrete various cytokines (interleukin-1 and interleukin-6) and growth factors (platelet-derived growth factor and transforming growth factor β) that promote smooth muscle cell (SMC) proliferation. Meanwhile, coagulation activation leads to thrombin formation, thus depositing polymeric fibrin. Thrombin directly or indirectly stimulates SMC proliferation through platelets-secreted platelet derived growth factor (PDGF). Neo-endothelium forms a layer of platelets and fibrin from the edge of the damaged area. The SMC in the inner layer undergoes phenotypic regulation from a static contraction state to a synthesis stage, similar to fibroblast migration towards the endometrium. The intima further thickens by secreting an extracellular matrix containing elastin, collagen, glycoprotein, and proteoglycan. Highly proliferative fibroblasts migrate from the adventitia to the endometrium and differentiate into myofibroblasts, promoting intima thickening. This process starts at the anastomotic site of the transplanted blood vessels and extends to the entire SVG over time (21).

As a result, aspirin combined with clopidogrel/ticagrelor DAPT has been widely used as a routine secondary preventive medication after CABG surgery. Several studies (since 1980s) have shown that aspirin can improve SVG patency (22). For instance, a meta-analysis with 25,728 patients showed that clopidogrel combined with aspirin can lower the incidence of early SVG failure [hazard ratio; 0.59 (95% CI, 0.43–0.82); *P* = 0.02] and a 30-day mortality rate (0.8% vs. 1.9%; *P* < 0.0001) (23). Compared with aspirin alone, CRYSSA trial showed that aspirin combined with clopidogrel can significantly reduce the occlusion rate of 12-month SVG in off-pump CABG patients (24). Furthermore, DACAB trial showed that ticagrelor + aspirin can increase SVG patency (ticagrelor + aspirin = 88.7% and aspirin alone = 76.5%, *P* < 0.001) one year after CABG surgery (10). However, 75.8% of patients in the DACAB trial received off-pump CABG, indicating that the results may mainly apply to off-pump CABG patients.

Two recent randomized controlled trials have also shown that the efficacy of off-pump CABG and on-pump CABG is not significantly different (2, 3). Extracorporeal circulation may impact the coagulation and platelet function of patients. To the best of our knowledge, this is the first clinical study to compare the effects of different DAPT strategies on SVG after off-pump CABG surgery. Patient recruitment was originally expected to be completed before April 2021. However, nearly half of the patients who met the recruitment criteria refused to participate due to various factors, such as unwillingness to sign informed consent and cooperate with follow-up. Additionally, COVID-19 affected patient recruitment and follow-up, and thus the study was completed much earlier. Nonetheless, this single-center, prospective, randomized, double-blind clinical controlled trial indicated that aspirin combined with ticagrelor DAPT after CABG can better maintain the patency of vein grafts one year after surgery. The findings indicated that ticagrelor may be a uni- and multivariate predictor for reducing occlusion of vein grafts.

Clopidogrel is a precursor drug metabolized by cytochrome P450 and an irreversible P2Y12 inhibitor. Clopidogrel can inhibit diphosphate-induced platelet aggregation, selectively inhibit platelet cyclooxygenase-1, and interrupt the formation of thromboxane A2 (17). Unlike clopidogrel, Ticagrelor reversibly binds directly, and requires two steps of cyp1-dependent metabolism (25), indicating that ticagrelor has a more substantial anti-platelet effect. Some scholars have in recent years conducted research on the effectiveness of aspirin combined with clopidogrel/ticagrelor DAPT strategies through randomized controlled trials with 147 CABG patients and shown that ticagrelor combined with clopidogrel does not significantly affect the patency of SVG (91.0% vs. 89.9% *P* = 0.751) (26). However, 69.4% of patients underwent on-pump CABG, indicating that the conclusions are not applicable to simple off-pump CABG patients (26). Additionally, more extensive sample studies are needed for further verification since this was a single-center study.

Previous PLATO studies showed that ticagrelor has a better therapeutic effect on acute coronary syndrome than clopidogrel. Furthermore, PLATO studies indicated that ticagrelor can significantly reduce the incidence of main cardiovascular death, myocardial infarction, and other serious adverse cardiovascular events during the one-year follow-up (9.8% vs. 11.7%, *P* < 0.001) (27). Ticagrelor also significantly reduced cardiovascular event mortality in the CABG subgroup evaluation (4.1% vs. 7.9%, *p* < 0.01) (8). Notably, increased postoperative LVD and decreased LVEF may be predictive factors for the prognosis (28). In this study, ticagrelor did not significantly impact postoperative LVD and LVEF, possibly due t small sample size. Moreover, there was low overall incidence of MACE events during the one-year follow-up, necessitaing further studies for validation.

Compared with aspirin alone, DATP can increase the risk of hemorrhage incidents (29). However, some scholars have shown that ticagrelor may have a higher risk of bleeding than clopidogrel (30). Overall, ticagrelor is usually limited to minor bleeding risk events, consistent with previous studies (31).

## Limitation

This was a single-center study. Although we enrolled participants according to the pre-determined sample size, the rate of venous graft stenosis/occlusion in Group C was lower than anticipated. In the initial design phase, we anticipated this possibility and planned to include more paticipants to ensure adequate statistical power. However, the recruitment was prematurely discontinued due to COVID-19. Consequently, further validation of these findings may require a larger-scale study. Additionally, this study did not perform clopidogrel genotype testing, patient genotype analysis, or gene-related analyses.

## Conclusion

Compared with clopidogrel, ticagrelor treatment after CABG can significantly reduce the occlusion rate of grafts. Furthermore, postoperative ticagrelor combined with aspirin DAPT can be a predictive factor for improving the patency of vein grafts.

## Data Availability

The datasets presented in this study can be found in online repositories. The names of the repository/repositories and accession number(s) can be found below: the study was registered at the Clinical Trial Center in China (Registration no. ChiCTR1900022390. Date of first registration: 09/04/2019).
